# GASP: Gapped Ancestral Sequence Prediction for proteins

**DOI:** 10.1186/1471-2105-5-123

**Published:** 2004-09-06

**Authors:** Richard J Edwards, Denis C Shields

**Affiliations:** 1 Bioinformatics Core, Clinical Pharmacology, Royal College of Surgeons in Ireland, 123 St Stephen's Green, Dublin 2, Ireland

## Abstract

**Background:**

The prediction of ancestral protein sequences from multiple sequence alignments is useful for many bioinformatics analyses. Predicting ancestral sequences is not a simple procedure and relies on accurate alignments and phylogenies. Several algorithms exist based on Maximum Parsimony or Maximum Likelihood methods but many current implementations are unable to process residues with gaps, which may represent insertion/deletion (indel) events or sequence fragments.

**Results:**

Here we present a new algorithm, GASP (Gapped Ancestral Sequence Prediction), for predicting ancestral sequences from phylogenetic trees and the corresponding multiple sequence alignments. Alignments may be of any size and contain gaps. GASP first assigns the positions of gaps in the phylogeny before using a likelihood-based approach centred on amino acid substitution matrices to assign ancestral amino acids. Important outgroup information is used by first working down from the tips of the tree to the root, using descendant data only to assign probabilities, and then working back up from the root to the tips using descendant and outgroup data to make predictions. GASP was tested on a number of simulated datasets based on real phylogenies. Prediction accuracy for ungapped data was similar to three alternative algorithms tested, with GASP performing better in some cases and worse in others. Adding simple insertions and deletions to the simulated data did not have a detrimental effect on GASP accuracy.

**Conclusions:**

GASP (Gapped Ancestral Sequence Prediction) will predict ancestral sequences from multiple protein alignments of any size. Although not as accurate in all cases as some of the more sophisticated maximum likelihood approaches, it can process a wide range of input phylogenies and will predict ancestral sequences for gapped and ungapped residues alike.

## Background

Predicting ancestral protein sequences from a multiple sequence alignment is a useful tool in bioinformatics [1]. Many evolutionary sequence analyses require such predictions in order to map substitutions to a particular lineage (*e.g. *[2,3]). In other situations, the predicted ancestral sequence alone may provide a more representative functional sequence than a simple consensus sequence constructed from an alignment.

Nevertheless, predicting ancestral sequences is not a simple procedure. It relies on a quality alignment plus an accurate – and correctly rooted – phylogenetic tree. Strict consensus methods are quick but can suffer from over-representation of larger clades of related sequences, which contribute more sequences to the consensus than more sparsely populated clades. Maximum Parsimony (MP) methods [4] overcome this problem by minimising mutational steps, rather than maximising agreement with the terminal sequences. MP, however, cannot distinguish between several equally parsimonious predictions. More sophisticated likelihood-based methods exist that can give probabilities for different ancestral sequences (*e.g. *[5-8]) and implementation such as CODEML [5] and FASTML [7] provide a good balance between speed and accuracy. However, many of these programs cannot predict ancestral sequences for columns of the alignment that have one or more gapped residues [9].

GASP (Gapped Ancestral Sequence Prediction) is an ancestral sequence prediction algorithm that is designed to handle gapped alignments of any size using a combination of MP and likelihood methods. Although probably not as accurate as some of the more sophisticated maximum likelihood approaches, it permits the estimation of ancestral states at residues that are gapped in any sequences of the alignment with comparable accuracy to that of ungapped residues.

## Implementation

### The GASP algorithm

#### Input

GASP uses input from three sources: a multiple sequence alignment (MSA); an accompanying phylogenetic tree in Newick format [10]; and a Point Altered Mutation (PAM) substitution probability matrix, such as that of Jones *et al. *1992 [11]. Sequences are read in from the alignment and node relationships established from the tree. The tree may be already rooted or rooted using GASP and must have branch lengths. Bootstrap values are not used by GASP but will be read if present. Sequences in the tree file can be represented by numbers (matching the order of the fasta alignment) or the first word of the sequence name. Details of the input formats can be found at: .

#### Output

**Figure 1:**
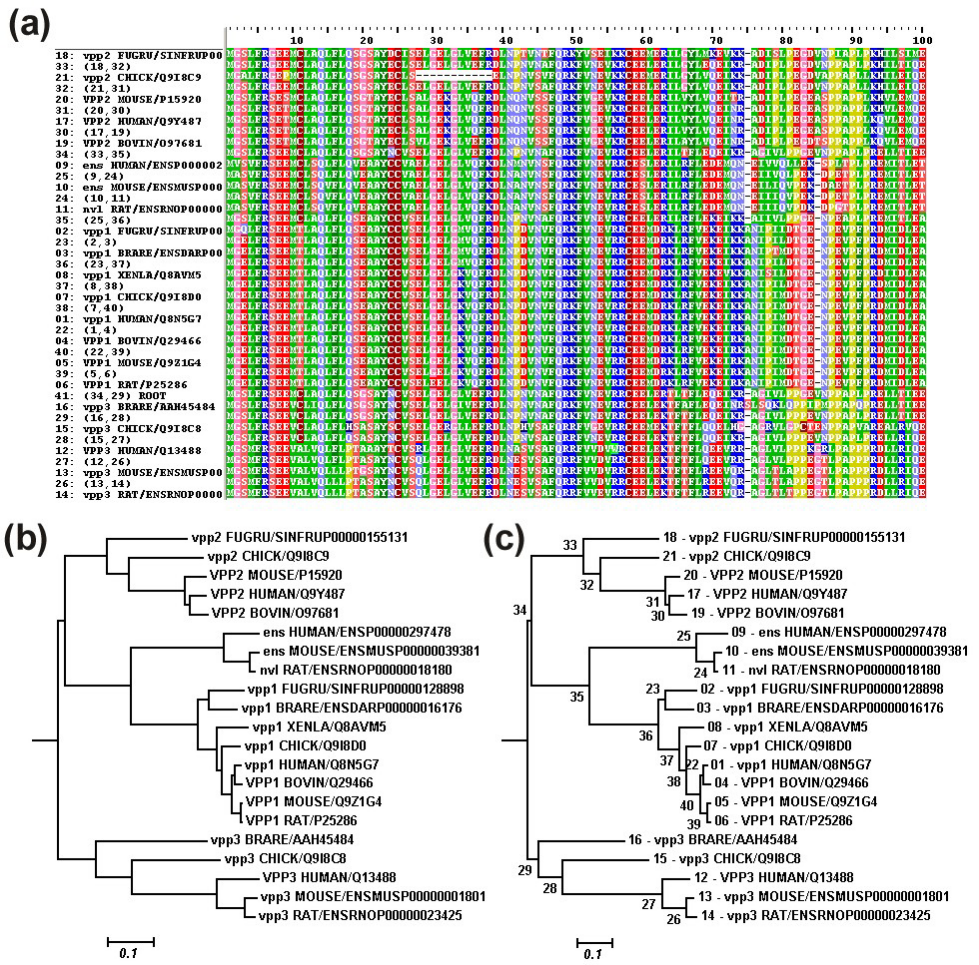
**Sample output from GASP. (a) **The first 100 columns of a typical GASP ancestral sequence prediction output. Sequence order matches the default vertical ordering in the tree files produced by GASP. **(b) **A rooted version of the input tree. Lengths of branches are those defined in the input file. **(c) **A new version of the input tree with nodes labelled and branch lengths recalculated based on ancestral sequence prediction. **Note. **Data is output in (a) fasta format and (b & c) Newick format but for visual clarity the file has been shown using (a) BioEdit [14] and (b & c) TreeExporer [12].

**Figure 2:**
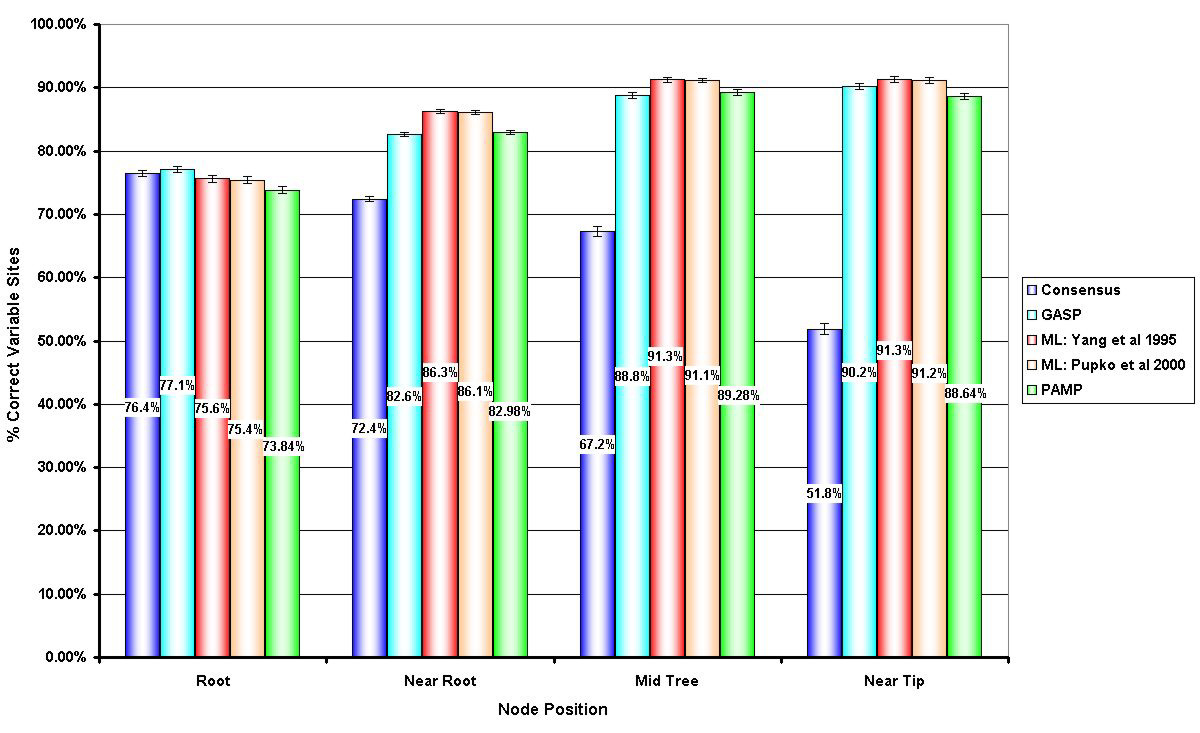
**Mean accuracies of methods using the 'PAM Variable Rates' Model. **Error Bars are Standard Errors. Percentage Accuracies are calculated for variable sites only (see text for details). The Percentage Accuracy for all sites is higher in all cases (Data not shown). The 'PAM Equal Rates' and 'Random Equal Rates' Models gave very similar results (Data Not Shown). Data shown includes only those phylogenies that did not crash CODEML (see text).

GASP outputs an alignment in fasta format with both input terminal sequences and predicted ancestral node sequences. Ancestral sequences can either be grouped together at the end of the file or interspersed throughout the terminal sequences to reflect the tree topology (Figure 1(a)). Three tree files are also output from GASP: (1) Newick format of the original input tree, rooted (Figure 1(b)); (2) A raw text version of the tree, with internal nodes numbered as for the output sequence file; (3) Newick format tree with node numbers instead of bootstrap values, which can be opened with K Tamura's TreeExplorer program [12] (Figure 1(c)). Branch lengths in this last file are replaced with the most likely PAM distance for a given branch, where PAM likelihoods for each branch are calculated as the product of each individual residue:


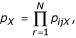


where *p*_*X *_is the likelihood for a PAM distance of *X *(see 'Ancestral sequences' below), *i *is the ancestral amino acid at position *r*,*j *is the descendant amino acid at position *r*, *p*_*ijX *_is the substitution probability of *i *to *j *in a PAM*X *matrix, and *N *is the number of residues in the alignment. Substitutions involving gaps are ignored in this calculation.

This allows a visual comparison between the branch lengths of the input phylogeny and the predicted branch lengths given the ancestral sequence predictions.

#### Gaps

If the MSA has gaps, GASP will first assign gap status to every residue at every node. Insertions and deletions are assumed to be equally likely, although a gap is assigned in the case of a tied probability (below). For each residue *r*, GASP starts at the tips and works deeper into the tree, assigning a probability of a gap at each node *n*, which is equal to the mean probability of a gap at the descendant nodes:


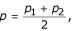


where *p *is the gap probability for residue *r *at node *n. p*_1 _and *p*_2 _are the gap probabilities for *r *at the two descendant nodes.

Terminal branches are given a probability of 1 if a gap is present or 0 if not. Once the root is reached, the gap status is fixed for the root. If the probability of a gap is greater than or equal to 0.5, residue *r *is fixed as a gap, otherwise *r *is fixed as a 'non-gap'. GASP then works back up the tree from the root, this time using the new ancestral gap probability and both descendant gap probabilities to recalculate the gap probability:


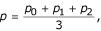


where *p*_0 _is the gap probability for *r *at the ancestral node.

As with the root, *r *is fixed as a gap if *p *≥ 0.5. This continues until all nodes are assigned as 'gap' or 'non-gap'.

#### Ancestral sequences

Once gaps are assigned, ancestral sequences are predicted in a similar fashion. Each residue *r *is assigned a probability for each amino acid at each node *n*. At the tips, *r *has a probability of 1 for the amino acid that is present in the MSA. GASP then works down the tree assigning probabilities based on the descendant nodes, branch lengths and a substitution matrix. By default, the PAM matrix of Jones *et al. *1992 [11] is used. This is a PAM**1 **matrix, which represents the probability that a given amino acid will be substituted by each other amino acid when the mean substitution rate is **1**/100 residues. To make a PAM*X *matrix, which represents a length of evolutionary time where a sequence will have undergone *X *substitutions per 100 residues, the PAM1 matrix is multiplied by itself *X*-1 times:


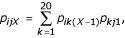


where *i *is the ancestral amino acid,*j *is the descendant amino acid, *k *is the 20 possible transitory amino acids, *p*_*ijX *_is the substitution probability of *i *to *j *in a PAM*X *matrix, *p*_*ik*(*X*-1) _is the substitution probability of *i *to *k *in a PAM(*X*-1) matrix and *p*_*kj*1 _is the substitution probability of *j *to *k *in a PAM1 matrix.

Unless the ancestral node has a gap (as calculated above) at position *r*, the ancestral probabilities for each amino acid are calculated for the two descendant branches individually, using a PAM*X *matrix, where *X *is 100 times the branch length as substitutions per site, *i.e. *a branch of 0.1 substitutions per site would use a PAM10 matrix:


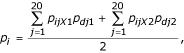


where *p*_*i *_is the probability of amino acid *i *at residue *r *of node *n*, *p*_*ijX*1 _and *p*_*ijX*2 _are the probabilities of substitution from amino acid *i *to each amino acid *j *in the appropriate PAM matrix for the two descendant branches, *p*_*dj*1 _and *p*_*dj*2 _are the probabilities of amino acid *j *being at position *r *at the two descendant nodes.

Once the root is reached, the most probable amino acid is fixed as the ancestral sequence. As with gaps, GASP then works back up the tree, using the fixed ancestral node amino acid and the descendant node probabilities to give new probabilities for each amino acid. The most probable amino acid is then fixed and the process continues until all residues and all nodes have a fixed sequence.

GASP is primarily designed for reasonably small trees (6–30 sequences), although there is no limit on input tree size. For larger trees, probabilities for each amino acid get very small near the root, which can lead to a heavy bias towards the fixed ancestral amino acid when GASP works back up the tree. To counter this GASP arbitrarily reduces any probabilities below a certain exclusion threshold (0.05 by default) to zero, thus reducing the slow accumulation of very unlikely amino acids.

#### Variations

To optimise the PAM matrices used for probability calculations, GASP uses the variable branch lengths read from the input phylogeny. There is also an option to fix the PAM distance used for all branches, which would allow the use of trees without branch lengths.

Assignment of ancestral amino acids with the GASP algorithm is achieved by combining data from the descendants of a given node *n *and its direct ancestor *n*_0_. This ancestor itself is heavily influenced by the descendants of *n *but also by the 'outgroup' to *n*, namely those sequences that are descendant to *n*_0 _but not to *n*. The outgroup information contained by the ancestral node *n*_0 _can be vital in determining the correct sequence for *n *when the descendants of *n *are variable. For this reason, the GASP algorithm will, by default, fix ancestral sequences as it moves back 'up' the tree from the root, increasing the relative weighting of the outgroup to the two descendants. Because there is a chance of the wrong amino acid sweeping back up the tree (especially if rare amino acid probabilities are allowed to accumulate by reducing the exclusion threshold), there is an option to use amino acid probabilities from the ancestral node in the last stage of GASP rather than giving the fixed amino acid an ancestral probability of 1. This option should be used with caution.

### Simulated datasets

#### Basic trees

To test the GASP algorithm, a number of artificial phylogenies were simulated. Because there is a practically limitless number of possible tree sizes (in both numbers of sequences and branch lengths) and phylogenies, it was decided to test the algorithm on a set of simulated phylogenies based on real phylogenies that formed a subset of those for which the algorithm was originally written. This set comprised 94 neighbour-joining trees of protein families. Each tree contained at least two subfamilies of at least 3 members each, giving in total between 6 and 127 sequences. (The program used to generate these simulated phylogenies is also available from the author for testing the algorithm on a different set of trees.)

Simulations started by creating a random protein sequence 100 amino acids long. Each residue was assigned an amino acid randomly as determined by the amino acid frequencies in all the human sequences of SwissProt-TrEMBL (Release 42) [13]. Sequences then evolved according to the template phylogeny. For each branch, residues were randomly substituted as described below until the total number of observed changes (ignoring multiple hits) equalled or exceeded the branch length of the phylogeny, which was not corrected for multiple hits. At this point, a new node was created with the new sequence and the tree split into two descendants. This proceeded until the whole phylogeny had been reconstructed. Each template tree seeded ten randomly simulated datasets. Algorithms were then given as input the simulated alignment and the parent phylogeny with 'real' branch lengths as calculated during simulation. (Note that PAML does not use these branch lengths.)

#### Substitution methods

Three substitution methods were used. In the first 'PAM Equal Rates' model, the PAM1 matrix of Jones *et al. *1992 [11] was used, giving variable rates of evolution for different amino acids and different substitution likelihoods. For comparison, a purely random substitution matrix was used where every amino acid had an equal probability of evolving into every other amino acid (the 'Random Equal Rates' method). Under these methods, different residues have similar rates of evolution. A further model was used based on the PAM1 method where residues had different probabilities of evolving, before amino acid-dependent PAM effects are considered. Under this 'PAM Variable Rates' model, 40% sites evolved at the 'normal' rate, 20% half-rate, 20% double rate, and 20% almost fixed (1/50 rate). Note that the observed branch lengths remain the same as the normal 'PAM Equal Rates' method but highly variable sites will be more likely to have multiple substitutions under the 'PAM Variable Rates' method.

#### Gapped data

Because one of the main advantages of GASP is its ability to deal with gaps, a second test dataset was generated from the 'PAM Equal Rates' set of trees, this time with gaps added. The addition of gaps was kept simple so that the exact same trees could be used for the gap analysis, allowing direct comparison of the results with gaps and without. (See Testing the GASP Algorithm, below.) To do this, gaps were limited to single insertion/deletion ('indel') events per column of the MSA, allowing them to overlay onto the existing simulated 'PAM Equal Rates' data. In addition, no indels occurring next to root were allowed as it is impossible to judge without an outgroup whether such an event would be an insertion or deletion.

To make the gaps, each residue *r *of the simulated sequences was considered in turn and had a probability of 50% of containing an indel. Gaps were all of length 1 (although two gaps may fall side by side, by chance). Although unrealistic for testing multiple alignment or phylogeny reconstruction programs, such a simplification is not a problem for ancestral sequence prediction as each residue is treated independently. The short gaps meant that, for the same total number of gapped residues, there is a higher diversity in the phylogenetic positioning of the indels.

Indels were placed randomly with respect to evolutionary time. Each node in the simulated data has an 'age', which is the number of rounds of potential substitution it took to complete the simulation after that node was formed. Each indel occurs at a random age *T *between the tip (age 0) and the oldest direct descendant node from the root. A random branch (not leading to root) is then selected for which the ancestral node is older than *T *and the descendant node is no older. This is the branch on which the indel occurred. The indel is randomly assigned as an insertion or deletion event with equal probability. If it is an insertion then the ancestral node plus all nodes outside the descendant clade have residue *r *replaced with a gap. If it is a deletion then the descendant node and all its descendants have residue *r *replaced with a gap.

## Results and discussion

### Testing the GASP algorithm

The simulated trees and alignments were run through the GASP algorithm. Because the 'real' sequence of each simulated node was known, it was then possible to determine the accuracy of GASP predictions. To test the different parts of the GASP algorithm, predictions were also made using modified GASP algorithms with parts of the model excluded.

Because prediction for invariant sites is trivial for all methods, the expectation is that accuracy is inversely related to the number of variable sites. Therefore, comparisons of methods are presented as a percentage of the variable sites. In this context 'variable sites' are defined independently for each node as those sites for which not all descendant nodes (including termini) have the same sequence as the ancestral node.

The simulated phylogenies are of different sizes. Considering all nodes of all trees would bias results towards the larger trees. To avoid this, each tree was arbitrarily reduced to four representative nodes:

1. 'Root' = The root of the tree.

2. 'Near Root' = A direct descendant node of the root.

3. 'Mid Tree' = A random node approx. midway in the tree.

4. 'Near Tip' = A direct ancestral node of a terminal sequence.

To determine whether the GASP algorithm was useful its performance was compared to a crude consensus sequence at each node. Where two amino acids were present at equal frequencies in a column of the MSA, the most frequent amino acid in the total MSA was selected for the ancestral sequence. GASP may be considered crude compared to some existing Maximum Likelihood approaches and so its performance was also compared to that of both ML algorithms implemented by the CODEML program from the PAML package [5], namely the marginal reconstruction algorithm of Yang *et al. *1995 [6] and the joint reconstruction algorithm of Pupko *et al. *2000 [7]. In addition, the MP method implemented in the PAMP program of PAML [9] was also tested for comparison.

The GASP model marginally out-performs all methods tested for constructing the ancestral sequence at the root of the tree (Figure 2). For all other representative node groups of the tree, GASP is comparable to the MP algorithm of PAMP but slightly inferior to both ML algorithms implemented in CODEML. PAMP is inferior to the ML methods at all levels of the tree. (In our hands, CODEML crashed in nearly 8% of cases. The problem was consistent and the troublesome input files crashed CODEML every time. However, there was no obvious difference between input files that presented CODEML with troubles and those that did not (Data not shown). To make a fair comparison of algorithms, data is only shown for datasets that did not cause CODEML to crash.)

**Figure 3:**
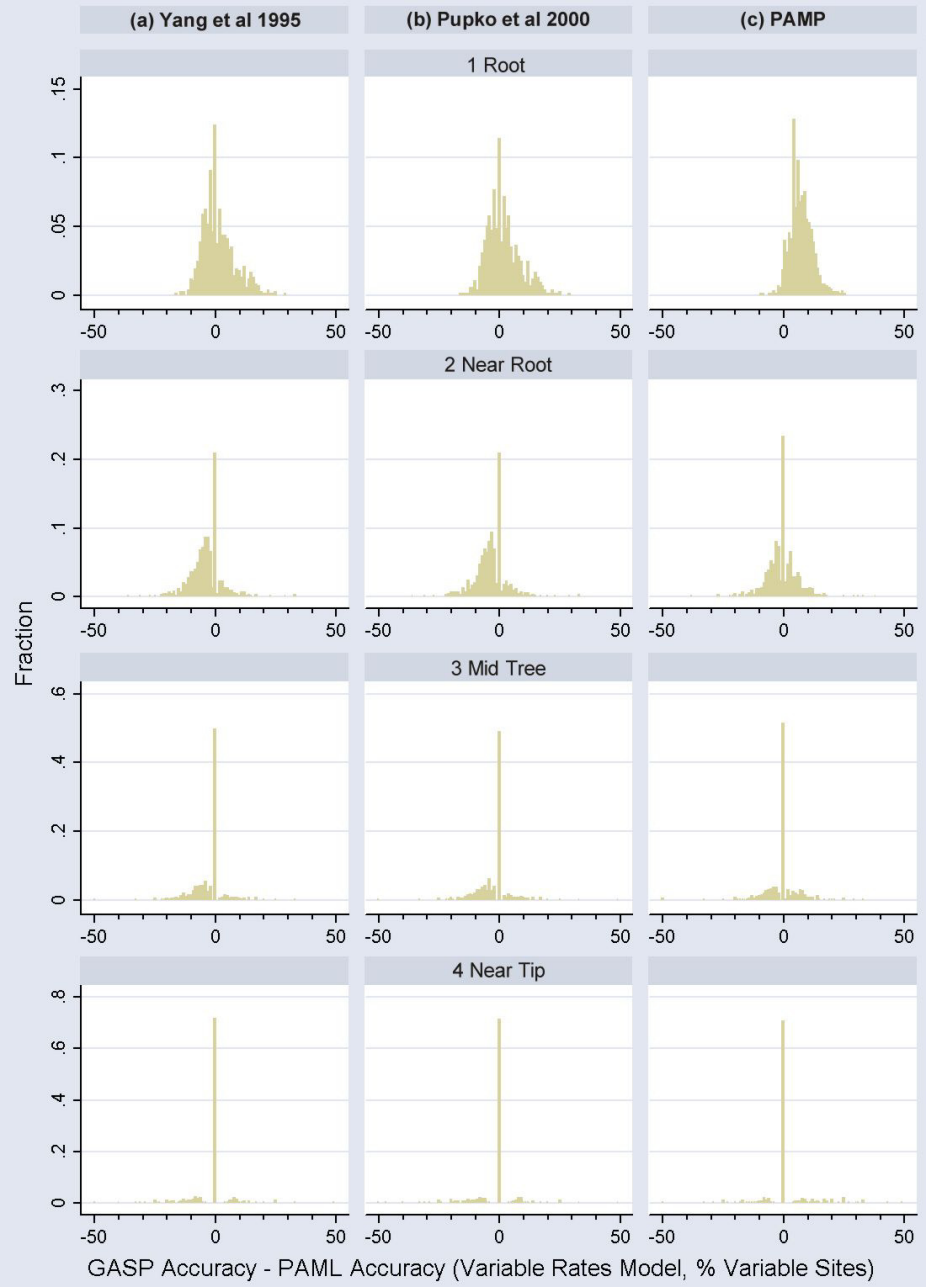
**Difference in prediction accuracies between GASP and three alternative algorithms. (a) **Yang *et al. *1995 ML [6], **(b) **Pupko *et al. *2000 [7] and **(c) **PAMP [9]. Percentage Accuracies are calculated for variable sites only and only those phylogenies that did not crash CODEML are shown (see text for details). Positive values indicate GASP is better than the other algorithm and negative values the reverse. Results for each tree depth are calculated separately. Values shown are for 'PAM Variable Rates' simulations only but the other evolutionary models give very similar results (Data not shown).

Although the ML algorithms of Yang *et al*. 1995 and Pupko *et al*. 2000 performed better overall for internal nodes, this difference was not seen for every node of every tree. At each level, GASP is sometimes better and sometimes worse than all three other algorithms (Figure 3). This is also true when comparing the three other algorithms with each other (Data not shown).

### GASP variants

Four individual elements of the GASP algorithm were explicitly tested by disabling each in turn and comparing the results to those generated by the complete algorithm. The four variants run were:

(a) using fixed PAM matrices rather than matrices derived from observed tree branch lengths.

(b) fixing ancestral sequences on initial pass towards root without a second pass back up the tree.

(c) no filtering of rare amino acid probabilities.

(d) using ancestral probabilities when working back up the tree rather than fixed ancestral amino acids.

Elements (a) and (b) were chosen for testing because they increase computational time, while (c) and (d) may not intuitively give the best results.

For the phylogenies used in these simulations, all four variants performed worse than the standard GASP algorithm (data not shown). Using a fixed PAM distance for all branches rather than approximating the PAM distance using tree branch lengths (a) gives an unfair weighting to long branches and thus increases the probability of substitutions that are, in reality, unlikely. Fixing ancestral sequences on the way 'down' the tree to the root (b) does not use any outgroup information and is therefore significantly worse at distinguishing between two or more amino acids with similar ancestral probabilities. Less intuitive is the effect of reducing low amino acid probabilities to zero (c) and using fixed ancestral sequences when recalculating amino acid probabilities using all three connected nodes (d). Indeed, excluding these two elements have a much smaller effect but still reduce the overall accuracy of the algorithm (data not shown).

Using fixed amino acids when working back up the tree increases the influence of the outgroup sequence. As was seen by the difference in accuracy between predictions at the root and nodes near the root (Figure 2), outgroup information is very important in predicting the correct sequence. (Predictions at the root are considerably weaker because there is no outgroup to help discriminate between alternative ancestral states.) Filtering out rare amino acids has a small effect in these trees but would be expected to have a larger effect in deeper trees. If rare probabilities are not removed then the most likely amino acid in each position will have an ever-diminishing likelihood, while highly unlikely ancestral sequences will find their probabilities ever-increasing. In very deep trees, this could result in probabilities being homogenised in the deep nodes. When fixed ancestral sequences are used to make predictions back up the tree, the fixed ancestral amino acid would potentially swamp the reduced probabilities in descendant nodes near the root, and sweep the root amino acid up the tree incorrectly. If this filtering is turned off when using larger trees, it is recommended that ancestral node probabilities be used instead of fixed ancestral sequences (*i.e. *combining (c) and (d)).

A final test was performed to compare the use of 'real' versus 'observed' branch lengths. (This was possible because the simulations kept track of not only what changes really occurred but also how many were 'visible', *i.e. *not correcting for multiple substitutions.) This is not testing the GASP algorithm *per se *but does provide information on the importance of using an accurate phylogeny construction algorithm. (The PAML package does not require pre-defined branch lengths and is therefore only affected by errors in supplied topology and not in branch lengths.) In many cases there was no difference. However, nearer to the root, using observed branch lengths rather than the real ones decreased prediction accuracy slightly. This decrease was correlated with total tree age (data not shown). This would imply that branch lengths corrected for multiple substitutions should be used for trees fed into the GASP algorithm, particularly with deep trees containing long branches.

### Gapped data

**Figure 4:**
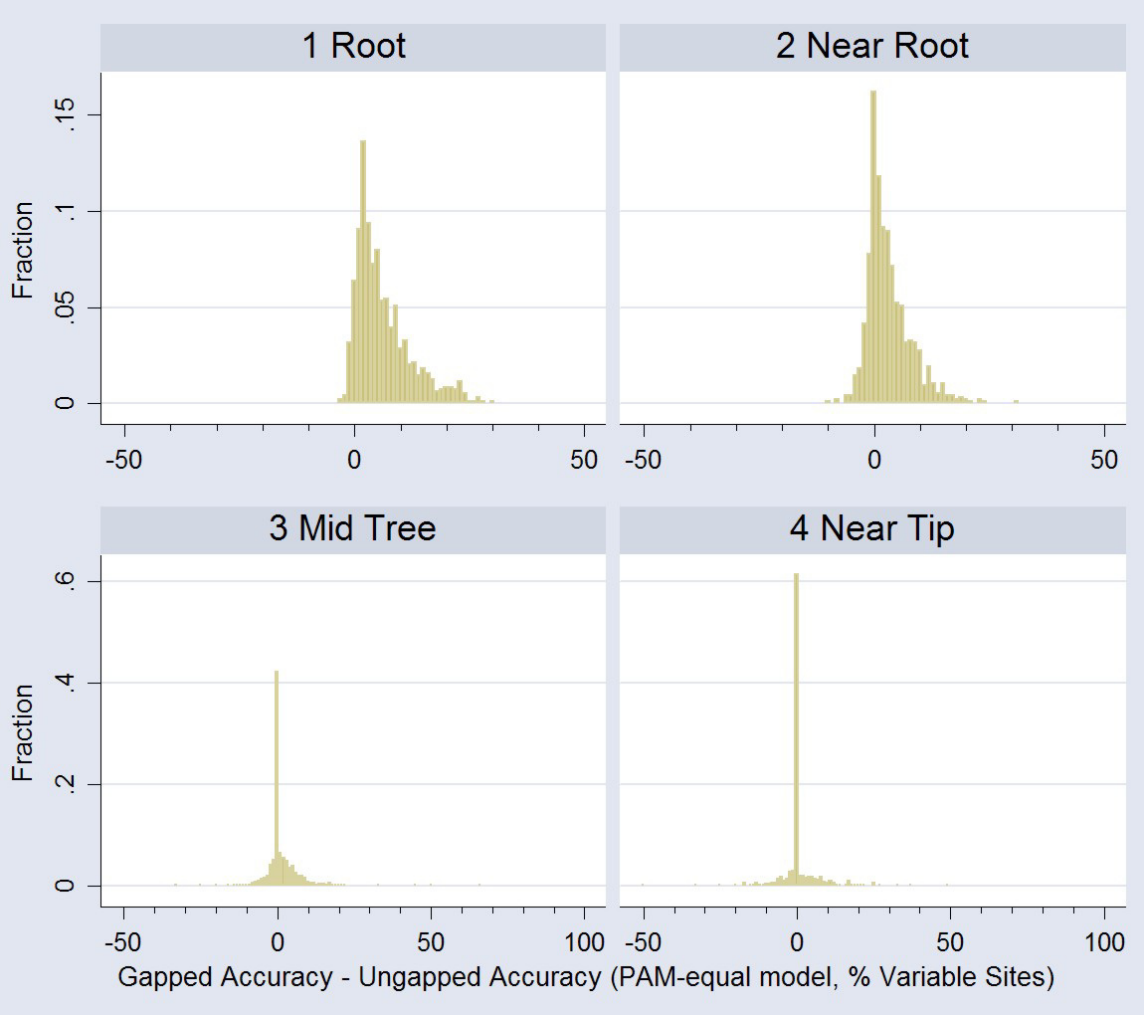
**Difference in GASP prediction accuracies of methods using gapped and ungapped 'PAM Equal Rates' simulations. **Percentage Accuracies are calculated for variable sites only (see text for details). Positive figures indicate that accuracies for the gapped dataset are higher than for the corresponding ungapped dataset. Results for each tree depth are calculated separately.

A central part of the GASP algorithm is its ability to handle gapped alignments. As expected, GASP correctly placed 100% of simple gaps used in this test. (Each column of the alignment has a maximum of one indel, which is descendant of the root branches.) To analyse the effect of gaps on prediction accuracy, pairwise comparisons were made between the gapped datasets and the corresponding ungapped simulations (Figure 4). As would be expected, some of the gapped data shows reduced prediction accuracy because, as with the root of the tree, there is no 'outgroup' information directly following an insertion event. In many situations, however, accuracy is increased. This is because a gap is easier to predict accurately (having only two states, present or absent) than an amino acid (which could be one of twenty). The Consensus method shows a similar pattern but with a smaller fraction of trees showing an increase in accuracy (Data not shown).

### DNA data

Although explicitly designed for use with protein sequence alignments and trees, it is relatively simple to convert GASP for use with nucleotide datasets. To do this, a new 'PAM matrix' should be created with substitutions probabilities for A, C, G and T only. This structure would allow the user to fit fairly complex substitution models, with different substitution probabilities for each pair of nucleotides. If the aligned sequence is coding DNA, however, it is highly recommended to use the protein sequences or a different algorithm such as those in the PAML package [5], as the adjusted PAM matrix would not take any consideration of codon positions.

## Conclusions

We have presented an algorithm for predicting ancestral sequences in gapped datasets. At the root of the tree, GASP marginally outperforms three existing algorithms implemented in the PAML package. For other nodes of the tree, however, the ML algorithms of CODEML [5-7] generally perform better than GASP, while PAMP [9] gives a similar performance. The main advantage of GASP is its ability to use gapped datasets. Simple indel patterns are accurately predicted by GASP and do not greatly decrease ancestral sequence prediction accuracy. The GASP algorithm can be reliably run on either Windows or UNIX platforms with no discernable instability.

For real life datasets, as for all evolutionary studies, predictions are dependent on the quality of input alignments. Gapped residues are, by their nature, often located in regions of evolutionary instability and therefore the interpretations of predictions at such sites require extra care. In many scenarios, however, gaps are introduced into alignments by the missing termini of fragment sequences. In these situations, the complete sequences that form the rest of the alignment may be very well aligned and so it is highly desirable to have an algorithm that can process the gaps introduced by the truncated sequences.

## Availability and requirements

**Project name: **GASP (Gapped Ancestral Sequence Prediction)

**Project home page: **

**Operating system(s): **Platform Independent. (Tested on PC (Windows 98/XP) and UNIX (Red Hat Linux 7.3))

**Programming language: **Perl.

**Other requirements: **None.

**License: **None.

**Any restrictions to use by non-academics: **Author's permission required.

## List of abbreviations

**GASP. **Gapped Ancestral Sequence Prediction.

**Indel. **Insertion or deletion event.

**ML. **Maximum Likelihood.

**MP. **Maximum Parsimony.

**MSA. **Multiple Sequence Alignment.

**PAM. **Point Accepted Mutation.

## Authors' contributions

RE conceived the algorithm, coded the Perl script, designed and performed the accuracy tests and statistical analyses, designed the phylogeny simulation method, generated the simulated datasets and drafted the manuscript. DS helped in the design of test simulations and in drafting the manuscript.
